# Apoptosis—50 Years after Its Discovery

**DOI:** 10.3390/biomedicines11041196

**Published:** 2023-04-18

**Authors:** Fernando Capela e Silva, Cecília M. P. Rodrigues

**Affiliations:** 1 Department of Medical and Health Sciences, School of Health and Human Development, MED—Mediterranean Institute for Agriculture, Environment and Development & CHANGE-Global Change and Sustainability Institute, Pólo da Mitra, University of Évora, 7000-671 Évora, Portugal; 2 Research Institute for Medicines (iMed.ULisboa), Faculty of Pharmacy, University of Lisbon, 1649-003 Lisboa, Portugal

Apoptosis is a programmed cell death routine that plays an essential role in several biological processes, namely, embryonic development, tissue homeostasis, and immune response [1,2]. Its discovery in the early 1970s by Kerr, Wyllie, and Currie [3] has revolutionized our understanding of cell death and its importance in various physiological and pathological conditions. This Special Issue commemorates the 50th anniversary of the discovery of apoptosis and aims to highlight specific recent advances in our understanding of this fundamental biological process. It includes a collection of four research articles and one review article that cover various aspects of apoptosis, including its molecular mechanisms, regulation, and implications in health and disease. The articles were contributed by dedicated researchers in the field who provide insights into the recent developments and future perspectives on the topic, and whose main results and contributions are summarized below.

Essential oils are natural plant compounds which are increasingly being used in the pharmaceutical and food industries due to their recognized biological properties, namely, antibacterial, antiseptic, antimutagenic, antioxidant, anti-inflammatory, and anticancer, and whose mechanisms of action include, among others, the induction of apoptosis. To safely ensure the use of eugenol and pulegone in humans and animals, Ribeiro-Silva et al. [4] evaluated, in mice, the effects of low doses (chronic toxicity) and high doses (acute toxicity) of pulegone, the main component of pennyroyal oil, and eugenol, present mainly in clove oil. At the end of the experiment, the authors observed, among other results, that the studied compounds induced behavioral changes in the animals; caused a decrease in body weight as well as food and water consumption; induced genetic damage; modulated serum levels of triglycerides and alanine aminotransferase; increased glutathione reductase at the highest dose; and induced some histopathological changes, including, at the highest dose, hepatocyte apoptosis. When analyzed together, the results suggest that eugenol and pulegone can exert beneficial or harmful effects, depending on the dose, whether applied alone or in combination.

Several studies have shown that dysregulation in the machinery of cell death by apoptosis is a hallmark of cancer. The observed alterations are responsible for both the development and progression of tumors, but also for their resistance to different therapies. Therefore, understanding the apoptotic components underlying their expression in carcinogenesis can help in tracking disease progression and in the development of new drugs and therapeutic approaches. Since the intratumoral heterogeneity of the prostate contributes to a limited response to treatments, the use of biomarkers is necessary to improve the prognostic survival of the patient. In this sense, Ionescu et al. [5] characterized, by immunohistochemistry, the tumor microenvironment (T lymphocyte infiltration, intratumoral CD34, and KI-67 expression) and, by flow cytometry, evaluated the biological mechanisms involved in the evolution of the prostate tumor process (cell cycle, cell proliferation by adhesion glycoproteins, apoptosis) in prostatic tumorigenesis using human tissue samples. The results of this study suggest that the analysis, by flow cytometry, of the cell cycle, apoptosis, and adhesion glycoproteins with a critical role in the proliferation of tumor cells; and the immunohistochemical expression of T cell infiltrates, Ki-67 and CD 34, are adequate and efficient techniques and, therefore, recommended for the evaluation of samples of prostatic tissue with hyperplasia and adenocarcinoma. These methods are worthy of exploration in the future.

Breast cancer is the most common type of cancer diagnosed in women and the second leading cause of cancer-related death in women. The need to use autophagy inhibitors as a type of therapy against cancer stems from the fact that it promotes the survival of tumor cells, protecting them from apoptosis. Autophagy exhibits tumor-promoting and -suppressive properties, so a simultaneous approach to apoptosis and autophagy could be an interesting and effective strategy to eliminate resistance to anticancer drugs. Using several breast cancer cell lines, Nguyen et al. [6] reported the suppressive role of SERTAD1 in apoptosis/anoikis, a protein widely accepted as an oncogene, and whose increased expression is found in multiple malignant conditions, also affecting cancer cell survival and tumorigenesis. The results showed that different types of cell death were induced in response to different doses of doxorubicin (Dox), presumably via lysosomal membrane permeabilization: a low dose of Dox activated autophagy, while a high dose of chemotherapy induced apoptosis. Targeting cancer cells with Dox and inhibiting autophagy increased apoptosis/anoikis, suggesting a new role for SERTAD1 in maintaining cell homeostasis.

The association of DNA damage and repair with aging processes is well known. Since this is a risk factor for the development of neurodegenerative diseases, the importance of understanding the role that DNA modifications play in the genesis of these diseases becomes evident. The histone variant H2AX is an essential component for nucleosome formation, chromatin remodeling, and DNA repair, and is also used in vitro as an assay for double-strand breaks in dsDNA. Cleaved caspase 3 (cCASP3) is one of several markers of apoptosis. The fully functional protease derives from an inactive precursor protein, becoming active by cleavage and, after a cascade of events, culminating in cell death by apoptosis. In this regard, Gionchiglia et al. [7], using untreated and X-ray irradiated mice, studied the association between CASP3 activation and H2AX γ phosphorylation in the aging brain. Using this model, the study demonstrated H2AX to be a reliable marker of DNA damage response (DDR) in vivo, as it associates with 53BP1 after irradiation. Many irradiated H2AX immunoreactive cells fail to repair their genetic material and undergo apoptosis with CASP3 cleavage, despite the incorporation of BrdU as an indicator of de novo DNA synthesis. On the other hand, the results also suggest that cells from the cerebral cortex, hippocampus, and SVZ/RMS/OB are subject to naturally occurring endogenous DNA damage, either in the form of direct double-strand breaks (DSBs) or as un-repaired single-strand breaks (SSBs) that are converted to DSBs. Incorporation of BrdU, with or without simultaneous expression of pHH3, can activate a DDR in postmitotic cortical neurons or cause an aberrant reentry into the cell cycle, with possible progression to a senescence-like state, in the neurogenic hippocampus and SVZ/RMS/ob.

Some recent work has suggested that apoptosis may be involved in neuropathic pain, a chronic pain state caused by primary injury or dysfunction of the nervous system, although the details of the underlying molecular mechanisms are not yet fully understood. The objective of the article by Ribeiro et al. [8] was to review the role of oxidative stress and neuroinflammation through the activation of cell signaling pathways that can lead to the destruction of the nerve terminal by apoptosis, as well as the clinical relevance of this knowledge. The potential discovery of new biomarkers and therapeutic targets may result in the development of more effective drugs and in progress in the treatment of chronic pain, namely neuropathic pain.

The discovery of apoptosis has had a profound impact on several fields of research, including, but not limited to, cancer biology, immunology, developmental biology, and neuroscience. It has led to the identification of critical genes and pathways involved in cell death and has paved the way for the development of new therapeutic strategies for various diseases [9]. This Special Issue celebrates the 50th anniversary of this landmark discovery and provides examples of specific advances in apoptosis and their significance in health and disease. We hope that this Special Issue will serve as a resource for researchers working in the field and inspire further progress in our understanding of apoptosis.
